# Supplementary material for: Heparin-based hydrogel scaffolding alters the transcriptomic profile and increases the chemoresistance of MDA-MB-231 triple-negative breast cancer cells
Source: Biomater Sci. 2020 Feb 13;8(10):2786–96. doi: 10.1039/c9bm01481k (PMC7497406; doi:10.1039/c9bm01481k)
Supplement: Supplementary file 2 [file BM-008-C9BM01481K-s002.zip › Supplementary File 4/HP-BhydrogelvControl/Pathways/my_analysis.Gsea.1545201079907/HALLMARK_EPITHELIAL_MESENCHYMAL_TRANSITION.html]

Details for gene set HALLMARK\_EPITHELIAL\_MESENCHYMAL\_TRANSITION[GSEA]

|  || Dataset | expr.class.cls#HYD\_versus\_CONTROL.class.cls#HYD\_versus\_CONTROL\_repos |
| Phenotype | class.cls#HYD\_versus\_CONTROL\_repos |
| Upregulated in class | CONTROL |
| GeneSet | HALLMARK\_EPITHELIAL\_MESENCHYMAL\_TRANSITION |
| Enrichment Score (ES) | -0.2137201 |
| Normalized Enrichment Score (NES) | -0.9971643 |
| Nominal p-value | 0.46153846 |
| FDR q-value | 0.4847154 |
| FWER p-Value | 0.994 |
Table: GSEA Results Summary

  

Fig 1: Enrichment plot: HALLMARK\_EPITHELIAL\_MESENCHYMAL\_TRANSITION      
 Profile of the Running ES Score & Positions of GeneSet Members on the Rank Ordered List

  

| PROBE | DESCRIPTION (from dataset) | GENE SYMBOL | GENE\_TITLE | RANK IN GENE LIST | RANK METRIC SCORE | RUNNING ES | CORE ENRICHMENT || 1 | COL16A1 | na |  |  | 8 | 4.252 | 0.0368 | No |
| 2 | COL7A1 | na |  |  | 428 | 2.435 | 0.0361 | No |
| 3 | COL1A1 | na |  |  | 499 | 2.347 | 0.0530 | No |
| 4 | COL8A2 | na |  |  | 580 | 2.237 | 0.0684 | No |
| 5 | COL5A1 | na |  |  | 935 | 1.921 | 0.0666 | No |
| 6 | SNTB1 | na |  |  | 1134 | 1.775 | 0.0718 | No |
| 7 | TAGLN | na |  |  | 1147 | 1.771 | 0.0866 | No |
| 8 | BASP1 | na |  |  | 1247 | 1.713 | 0.0964 | No |
| 9 | TPM2 | na |  |  | 1256 | 1.709 | 0.1110 | No |
| 10 | ECM2 | na |  |  | 1285 | 1.696 | 0.1244 | No |
| 11 | ID2 | na |  |  | 1383 | 1.637 | 0.1336 | No |
| 12 | COL4A2 | na |  |  | 1514 | 1.570 | 0.1405 | No |
| 13 | PVR | na |  |  | 1551 | 1.551 | 0.1522 | No |
| 14 | ACTA2 | na |  |  | 1554 | 1.549 | 0.1657 | No |
| 15 | VEGFC | na |  |  | 1636 | 1.517 | 0.1747 | No |
| 16 | GADD45B | na |  |  | 1972 | 1.375 | 0.1692 | No |
| 17 | JUN | na |  |  | 1992 | 1.366 | 0.1801 | No |
| 18 | COL5A2 | na |  |  | 2317 | 1.233 | 0.1739 | No |
| 19 | GADD45A | na |  |  | 2424 | 1.200 | 0.1788 | No |
| 20 | EFEMP2 | na |  |  | 2427 | 1.200 | 0.1892 | No |
| 21 | MYLK | na |  |  | 2690 | 1.120 | 0.1853 | No |
| 22 | SLC6A8 | na |  |  | 3116 | 1.006 | 0.1718 | No |
| 23 | ADAM12 | na |  |  | 3349 | 0.953 | 0.1680 | No |
| 24 | LOXL1 | na |  |  | 3490 | 0.920 | 0.1687 | No |
| 25 | GPC1 | na |  |  | 3500 | 0.918 | 0.1762 | No |
| 26 | LAMA2 | na |  |  | 3545 | 0.908 | 0.1819 | No |
| 27 | COL6A3 | na |  |  | 3618 | 0.889 | 0.1859 | No |
| 28 | TNFRSF12A | na |  |  | 3645 | 0.883 | 0.1922 | No |
| 29 | HTRA1 | na |  |  | 3847 | 0.842 | 0.1891 | No |
| 30 | COL4A1 | na |  |  | 3868 | 0.840 | 0.1954 | No |
| 31 | NID2 | na |  |  | 4308 | 0.750 | 0.1789 | No |
| 32 | PMEPA1 | na |  |  | 4320 | 0.748 | 0.1849 | No |
| 33 | APLP1 | na |  |  | 4385 | 0.735 | 0.1879 | No |
| 34 | PLOD3 | na |  |  | 4440 | 0.726 | 0.1915 | No |
| 35 | ITGB5 | na |  |  | 4750 | 0.668 | 0.1811 | No |
| 36 | OXTR | na |  |  | 4918 | 0.638 | 0.1779 | No |
| 37 | MCM7 | na |  |  | 5533 | 0.548 | 0.1505 | No |
| 38 | FBLN1 | na |  |  | 5556 | 0.544 | 0.1541 | No |
| 39 | LAMA3 | na |  |  | 5687 | 0.527 | 0.1519 | No |
| 40 | BMP1 | na |  |  | 5841 | 0.508 | 0.1483 | No |
| 41 | FSTL3 | na |  |  | 6052 | 0.480 | 0.1414 | No |
| 42 | QSOX1 | na |  |  | 6228 | 0.455 | 0.1362 | No |
| 43 | LRP1 | na |  |  | 6291 | 0.446 | 0.1369 | No |
| 44 | PTX3 | na |  |  | 6358 | 0.435 | 0.1372 | No |
| 45 | BDNF | na |  |  | 6575 | 0.406 | 0.1294 | No |
| 46 | CTGF | na |  |  | 6894 | 0.371 | 0.1160 | No |
| 47 | TNFAIP3 | na |  |  | 6980 | 0.364 | 0.1147 | No |
| 48 | IL6 | na |  |  | 7244 | 0.333 | 0.1038 | No |
| 49 | TGFBR3 | na |  |  | 7615 | 0.290 | 0.0869 | No |
| 50 | ELN | na |  |  | 7690 | 0.284 | 0.0856 | No |
| 51 | CXCL1 | na |  |  | 7814 | 0.270 | 0.0815 | No |
| 52 | COL12A1 | na |  |  | 7870 | 0.265 | 0.0809 | No |
| 53 | COL1A2 | na |  |  | 8100 | 0.246 | 0.0710 | No |
| 54 | MYL9 | na |  |  | 8250 | 0.226 | 0.0652 | No |
| 55 | COL6A2 | na |  |  | 8304 | 0.219 | 0.0643 | No |
| 56 | FZD8 | na |  |  | 8335 | 0.215 | 0.0646 | No |
| 57 | NOTCH2 | na |  |  | 8394 | 0.208 | 0.0634 | No |
| 58 | FBN1 | na |  |  | 8665 | 0.184 | 0.0508 | No |
| 59 | TNC | na |  |  | 9035 | 0.149 | 0.0328 | No |
| 60 | SNAI2 | na |  |  | 9111 | 0.139 | 0.0301 | No |
| 61 | LOXL2 | na |  |  | 9224 | 0.126 | 0.0253 | No |
| 62 | RHOB | na |  |  | 9250 | 0.124 | 0.0250 | No |
| 63 | LAMC1 | na |  |  | 9285 | 0.120 | 0.0243 | No |
| 64 | CALD1 | na |  |  | 9689 | 0.077 | 0.0038 | No |
| 65 | FUCA1 | na |  |  | 9835 | 0.060 | -0.0033 | No |
| 66 | PDGFRB | na |  |  | 9922 | 0.051 | -0.0073 | No |
| 67 | TGFB1 | na |  |  | 9937 | 0.048 | -0.0076 | No |
| 68 | TNFRSF11B | na |  |  | 10126 | 0.025 | -0.0173 | No |
| 69 | RGS4 | na |  |  | 10151 | 0.021 | -0.0184 | No |
| 70 | MATN3 | na |  |  | 10494 | -0.004 | -0.0363 | No |
| 71 | TPM1 | na |  |  | 10625 | -0.019 | -0.0429 | No |
| 72 | MATN2 | na |  |  | 10705 | -0.030 | -0.0468 | No |
| 73 | MAGEE1 | na |  |  | 10811 | -0.039 | -0.0520 | No |
| 74 | LRRC15 | na |  |  | 10849 | -0.046 | -0.0535 | No |
| 75 | SDC1 | na |  |  | 11066 | -0.073 | -0.0642 | No |
| 76 | ENO2 | na |  |  | 11078 | -0.075 | -0.0641 | No |
| 77 | CD44 | na |  |  | 11081 | -0.075 | -0.0636 | No |
| 78 | SERPINE2 | na |  |  | 11398 | -0.100 | -0.0793 | No |
| 79 | PLAUR | na |  |  | 11509 | -0.112 | -0.0841 | No |
| 80 | THBS2 | na |  |  | 11641 | -0.131 | -0.0898 | No |
| 81 | SERPINH1 | na |  |  | 11747 | -0.145 | -0.0941 | No |
| 82 | LAMC2 | na |  |  | 11847 | -0.157 | -0.0979 | No |
| 83 | MMP3 | na |  |  | 11975 | -0.170 | -0.1031 | No |
| 84 | ABI3BP | na |  |  | 11996 | -0.172 | -0.1026 | No |
| 85 | SLIT2 | na |  |  | 12028 | -0.177 | -0.1027 | No |
| 86 | CAP2 | na |  |  | 12189 | -0.199 | -0.1094 | No |
| 87 | PDLIM4 | na |  |  | 12197 | -0.199 | -0.1080 | No |
| 88 | PPIB | na |  |  | 12340 | -0.219 | -0.1135 | No |
| 89 | MEST | na |  |  | 12346 | -0.220 | -0.1119 | No |
| 90 | PLOD1 | na |  |  | 12612 | -0.235 | -0.1237 | No |
| 91 | DKK1 | na |  |  | 12791 | -0.256 | -0.1308 | No |
| 92 | FAS | na |  |  | 12828 | -0.261 | -0.1304 | No |
| 93 | IL32 | na |  |  | 12998 | -0.283 | -0.1368 | No |
| 94 | ITGA5 | na |  |  | 13121 | -0.302 | -0.1406 | No |
| 95 | FSTL1 | na |  |  | 13295 | -0.325 | -0.1468 | No |
| 96 | VCAN | na |  |  | 13320 | -0.327 | -0.1452 | No |
| 97 | TIMP3 | na |  |  | 13429 | -0.342 | -0.1479 | No |
| 98 | SDC4 | na |  |  | 13470 | -0.349 | -0.1469 | No |
| 99 | INHBA | na |  |  | 13523 | -0.357 | -0.1465 | No |
| 100 | SAT1 | na |  |  | 13558 | -0.362 | -0.1452 | No |
| 101 | WIPF1 | na |  |  | 13585 | -0.367 | -0.1433 | No |
| 102 | ECM1 | na |  |  | 13763 | -0.397 | -0.1491 | No |
| 103 | FERMT2 | na |  |  | 13772 | -0.398 | -0.1461 | No |
| 104 | TGM2 | na |  |  | 13799 | -0.403 | -0.1439 | No |
| 105 | WNT5A | na |  |  | 13835 | -0.411 | -0.1421 | No |
| 106 | GPX7 | na |  |  | 14117 | -0.430 | -0.1531 | No |
| 107 | MMP1 | na |  |  | 14272 | -0.457 | -0.1572 | No |
| 108 | COPA | na |  |  | 14280 | -0.459 | -0.1536 | No |
| 109 | PRSS2 | na |  |  | 14392 | -0.476 | -0.1552 | No |
| 110 | CDH11 | na |  |  | 14430 | -0.480 | -0.1530 | No |
| 111 | TIMP1 | na |  |  | 14560 | -0.499 | -0.1554 | No |
| 112 | PCOLCE | na |  |  | 14708 | -0.524 | -0.1585 | No |
| 113 | IL15 | na |  |  | 15098 | -0.579 | -0.1738 | No |
| 114 | PCOLCE2 | na |  |  | 15318 | -0.621 | -0.1799 | No |
| 115 | SPOCK1 | na |  |  | 15503 | -0.651 | -0.1839 | No |
| 116 | CAPG | na |  |  | 15558 | -0.662 | -0.1809 | No |
| 117 | EMP3 | na |  |  | 15769 | -0.696 | -0.1858 | No |
| 118 | IGFBP3 | na |  |  | 16184 | -0.784 | -0.2007 | No |
| 119 | ITGB3 | na |  |  | 16433 | -0.839 | -0.2064 | Yes |
| 120 | TFPI2 | na |  |  | 16544 | -0.865 | -0.2046 | Yes |
| 121 | CADM1 | na |  |  | 16551 | -0.866 | -0.1973 | Yes |
| 122 | PFN2 | na |  |  | 16639 | -0.883 | -0.1941 | Yes |
| 123 | DAB2 | na |  |  | 16686 | -0.894 | -0.1887 | Yes |
| 124 | CTHRC1 | na |  |  | 16722 | -0.905 | -0.1826 | Yes |
| 125 | TGFBI | na |  |  | 16797 | -0.927 | -0.1784 | Yes |
| 126 | PRRX1 | na |  |  | 16827 | -0.933 | -0.1717 | Yes |
| 127 | PTHLH | na |  |  | 16928 | -0.964 | -0.1686 | Yes |
| 128 | ITGAV | na |  |  | 16980 | -0.980 | -0.1627 | Yes |
| 129 | VCAM1 | na |  |  | 16995 | -0.984 | -0.1548 | Yes |
| 130 | NNMT | na |  |  | 17005 | -0.985 | -0.1466 | Yes |
| 131 | GLIPR1 | na |  |  | 17082 | -1.010 | -0.1418 | Yes |
| 132 | TPM4 | na |  |  | 17203 | -1.053 | -0.1389 | Yes |
| 133 | SPARC | na |  |  | 17223 | -1.058 | -0.1306 | Yes |
| 134 | ITGA2 | na |  |  | 17270 | -1.074 | -0.1236 | Yes |
| 135 | PLOD2 | na |  |  | 17330 | -1.095 | -0.1171 | Yes |
| 136 | LOX | na |  |  | 17344 | -1.100 | -0.1082 | Yes |
| 137 | GEM | na |  |  | 17475 | -1.142 | -0.1050 | Yes |
| 138 | SCG2 | na |  |  | 17637 | -1.216 | -0.1028 | Yes |
| 139 | CALU | na |  |  | 17764 | -1.268 | -0.0983 | Yes |
| 140 | SFRP1 | na |  |  | 17943 | -1.343 | -0.0959 | Yes |
| 141 | AREG | na |  |  | 18122 | -1.456 | -0.0925 | Yes |
| 142 | LGALS1 | na |  |  | 18128 | -1.458 | -0.0800 | Yes |
| 143 | DPYSL3 | na |  |  | 18168 | -1.479 | -0.0691 | Yes |
| 144 | VIM | na |  |  | 18288 | -1.557 | -0.0617 | Yes |
| 145 | NT5E | na |  |  | 18421 | -1.652 | -0.0542 | Yes |
| 146 | PMP22 | na |  |  | 18769 | -1.973 | -0.0551 | Yes |
| 147 | SGCB | na |  |  | 18835 | -2.064 | -0.0405 | Yes |
| 148 | EDIL3 | na |  |  | 18897 | -2.163 | -0.0247 | Yes |
| 149 | GJA1 | na |  |  | 18902 | -2.173 | -0.0059 | Yes |
| 150 | CD59 | na |  |  | 19031 | -2.444 | 0.0088 | Yes |
Table: GSEA details [plain text format]

  

Fig 2: HALLMARK\_EPITHELIAL\_MESENCHYMAL\_TRANSITION      
 Blue-Pink O' Gram in the Space of the Analyzed GeneSet

  

Fig 3: HALLMARK\_EPITHELIAL\_MESENCHYMAL\_TRANSITION: Random ES distribution      
 Gene set null distribution of ES for **HALLMARK\_EPITHELIAL\_MESENCHYMAL\_TRANSITION**
